# Au nanoparticle-based sensor for apomorphine detection in plasma

**DOI:** 10.3762/bjnano.6.228

**Published:** 2015-11-26

**Authors:** Chiara Zanchi, Andrea Lucotti, Matteo Tommasini, Sebastiano Trusso, Ugo de Grazia, Emilio Ciusani, Paolo M Ossi

**Affiliations:** 1 Dipartimento di Energia, Politecnico di Milano, Via Ponzio 34/3, 20133 Milano, Italy; 2 Dipartimento di Chimica, Materiali e Ingegneria Chimica ‘Giulio Natta’, Politecnico di Milano, Piazza Leonardo da Vinci 32, 20133 Milano, Italy; 3 CNR-IPCF, Istituto per i Processi Chimico-Fisici del CNR, V.le. F. S. D’Alcontres 37, 98158 Messina, Italy; 4 Laboratorio di Patologia Clinica e Genetica Medica, Fondazione IRCCS Istituto Neurologico Carlo Besta, Via Celoria 11, 20133 Milano, Italy

**Keywords:** apomorphine, Au NPs, nano-roughened films, pulsed laser deposition, self-assembled films, SERS

## Abstract

Artificially roughened gold surfaces with controlled nanostructure produced by pulsed laser deposition have been investigated as sensors for apomorphine detection aiming at clinical application. The use of such gold surfaces has been optimized using aqueous solutions of apomorphine in the concentration range between 3.3 × 10^−4^ M and 3.3 × 10^−7^ M. The experimental parameters have been investigated and the dynamic concentration range of the sensor has been assessed by the selection of two apomorphine surface enhanced Raman scattering (SERS) peaks. The sensor behavior used to detect apomorphine in unfiltered human blood plasma is presented and discussed.

## Introduction

In recent years, the analytical applications of Raman spectroscopy and its enhanced variant employing plasmonic media, the surface enhanced Raman scattering (SERS) effect, have significantly grown [1–7]. These applications have been fostered by the availability of noble metal nanostructures, which are either intentionally fabricated with the aim of optimizing the signal intensity and reproducibility [2–3] or carefully functionalized for the selective and sensitive detection of, for example, tumor cells [4]. Combining the SERS effect with scanning probe microscopy techniques (tip-enhanced Raman spectroscopy, TERS), molecular information can be obtained with high spatial resolution to show differences in the local chemical composition of, for example, a single tobacco virus [8].

The various SERS substrates reported in the literature and the associated experimental approaches to detect the spectroscopic signal attest the significant interest for SERS as a powerful, molecule sensitive, analytical technique. For instance, gold colloids synthesized by laser ablation in liquids, suitably functionalized and tagged with Raman reporters, have effectively revealed specific biomolecules, even in chemically complex environments such as cells [9–12]. On the other hand, silver and gold colloids produced by chemical routes can be effectively used to detect specific analytes of biomedical interest [13–16]. They can also reveal subtle chemical differences in samples from controls and from patients for specific cancer types [17]. Nanofabricated plasmonic substrates bear interesting perspectives for the detection of low concentration analytes in biomedical scenarios [18]. The interest in the use of plasmonics for enhancing optical spectroscopy techniques is certainly growing in the scientific community. This is testified by the recent birth of a series of international conferences on enhanced spectroscopy techniques [19–20].

In this work, we report on the use of gold nanoparticle (NP) arrays deposited on standard glass substrates to quantitatively detect apomorphine (APO) aiming at the realization of a biomedical sensor. APO has been long used in the treatment of patients with complicated Parkinson’s disease (PD). Indeed one of the challenging problems in the management of PD is the development of severe, long-term motor complications that oral medication cannot adequately control at tolerable doses. Presently, APO delivered via continuous subcutaneous infusion is one of the viable alternatives to oral medication in such patients. Adjusting the APO concentration as it fluctuates with time is critical and is monitored via standardized clinical observations and tests of motor condition [21]. Standard chemical analysis (HPLC) on blood samples from the patient is an alternative to obtain the APO concentration. Au NPs were synthesized by pulsed laser ablation of a metal target in inert gas at high pressure. With respect to free expansion in vacuum, the ambient gas modifies the expansion of the plasma plume, consisting of species ablated from the target surface. Depending on the nature of the gas and the pressure, significant volume contraction of the propagating plume can be achieved, providing ideal conditions to synthesize NPs. By changing the process parameters (in particular, the number of laser pulses, laser pulse energy and spot size on the target, gas characteristics and pressure), NPs with different sizes are produced and NP arrays with different degrees of surface coverage on the substrate (corresponding to strongly differentiated morphologies) are obtained in a finely controlled way [22–25]. Such nanostructured, artificially roughened surfaces are characterized by plasmonic properties, such as the frequency and width of the plasmon resonance, that can be tailored exploring wide intervals [26]. This makes them suitable for use as substrates for SERS, and in particular, to detect exiguous amounts of analyte with low-intensity Raman signatures or that are hindered by competitive fluorescence. A thorough exploration of the morphology versus performance relation for Au substrates with strongly different morphologies tested against various analytes indicates that films consisting of NPs mutually assembled to form islands separated by channels give the best SERS signal enhancements. The analytes tested range from a straightforward SERS label (RH6G) to organic dyes of interest in art conservation (Alizarin, Purpurin) to small proteins (lysozime). These results also indicate that there is a trade-off between the size of the analyte and the average size of the inter-island channels where the analyte is pinned to the substrate [27]. SERS spectra obtained with such substrates are remarkably reproducible and spatially uniform [28–29] thus they appear suitable to be used to rapidly measure the concentration of drugs in blood plasma. We note that, although in principle such substrates can be functionalized, for simplicity, we avoid this strategy since APO can be chemisorbed on the Au surface, giving sizeable SERS features.

## Experimental

### Production of gold substrates and SEM characterization

The substrates were prepared in a vacuum chamber at a base pressure lower than 10^−4^ Pa. A KrF excimer laser (λ = 248 nm, pulse width 25 ns, repetition rate 10 Hz) was focused onto a pure gold target (99.99%) mounted on a rotating holder. The substrates were deposited on pieces of Corning Glass 5049, or Si(100), placed at a distance of 35 mm from the target and held at room temperature. The target holder was rotated to avoid target surface cratering under repetitive ablation. The ablation was performed in an Ar atmosphere at a pressure of 100 Pa. The number of laser pulses was fixed at 10,000. The laser fluence was kept constant at 2.0 J/cm^2^. The sample morphology was observed by scanning electron microscopy (SEM) using a Zeiss Supra 40 field ion microscope.

### Preparation of APO solutions

For the preparation of APO solutions at different concentrations, the commercially available Apofin solution for subcutaneous injection (Chiesi Farmaceutici, Parma, Italy) was used. The commercial drug (3 mL) contains 30 mg of apomorphine hydrochloride (*M*_w_ = 303.78332 g/mol) as the active ingredient, corresponding to a molar concentration of 3.3 × 10^−2^ M of R-apomorphine, plus sodium metabisulfite and hydrochloric acid as excipients.

The APO solutions were obtained by suitable dilution of the commercial drug with sterile water for injection (B. Braun) to reach the final concentrations of 100, 10, 1 and 0.1 µg/mL (i.e., from 3.3 × 10^−4^ M to 3.3 × 10^−7^ M). The pH values of all solutions were in the range between 5.5 and 6.5, as determined by indicator paper.

### SERS of APO in aqueous solution and data processing

The gold SERS-active substrates were dipped in freshly prepared APO solutions (1 mL of each solution in a glass vial) for a fixed time and air-dried before acquiring SERS spectra. Three different dipping times were considered, namely 2, 5 and 10 min, in order to evaluate the best contact time for the solution and the substrate.

The gold substrates subjected to the dipping and drying procedure were observed with an Olympus BX41 microscope using a 50× Olympus objective (NA = 0.75) coupled to the spectrometer, and different points for each sample were chosen for SERS measurements. All SERS spectra were collected by a Jobin-Yvon LabRAM HR800 Raman spectrometer with a solid-state laser (Laser XTRA, Toptica Photonics) operating at 785 nm, equipped with a 600 grooves mm^−1^ grating and a Peltier-cooled CCD detector. Notch filters for 785 nm were used to suppress the Rayleigh scattering. The laser was focused on the sample through the same 50× Olympus objective (NA = 0.75), with a nominal laser power irradiation at the samples of 1 mW, over a spot size of about 1 µm, and an exposure time of 2 to 30 s (average of 2 accumulations). The spectra were recorded in the region between 900 and 1800 cm^−1^, where the most intense Raman features of apomorphine lie [30].

Two SERS peaks, namely at 1353 cm^−1^ and 1515 cm^−1^, were selected as intense, stable and reproducible markers for APO detection in aqueous solutions. The corrected areas under each peak were evaluated by manual selection of the baseline using Omnic 8 software (Thermo Fisher Scientific, Inc.). From repeated measurements at different spots, a mean value and standard deviation from the mean were calculated and studied as a function of drug concentration in order to assess the dynamic range of concentration of the sensor. Different fits were applied to the data (Sigma Plot, Systat Software, Inc., USA) to describe the concentration dependence in the range between 1 and 100 µg/mL.

The SERS peak areas were normalized with respect to the peak area for the highest concentration studied (i.e., 100 µg/mL), and plotted as a function of the logarithm of the APO concentration, in order to compare experimental data at different dipping times. A linear regression was applied to the data sets.

### SERS of APO in blood plasma

In order to prove the feasibility of the method for the detection of APO in real samples, some initial trials were carried out on unfiltered blood plasma from a healthy volunteer (Fondazione I.R.C.C.S. Istituto Neurologico Carlo Besta, Milano, Italy). The samples of plasma were supplemented with apofin to produce the desired concentrations of 200, 20, and 2 µg/mL of APO in plasma.

Starting from the lowest APO concentration (2 µg/mL), one SERS sensor was dipped for 5 min in 1 mL of plasma and dried in air before acquisition of the spectra. The experimental procedure was repeated, dipping the same sensor in plasma containing APO at 20 µg/mL for 2 min. The sensor was finally dipped for 2 min in the most concentrated sample (200 µg/mL), air-dried and measured. All spectra were collected in the 900–1800 cm^−1^ spectral range with exposure times of 5, 10, 30 and 60 s (average of 2 accumulations).

## Results and Discussion

### Morphology of nanostructured gold substrates

**Figure 1:**
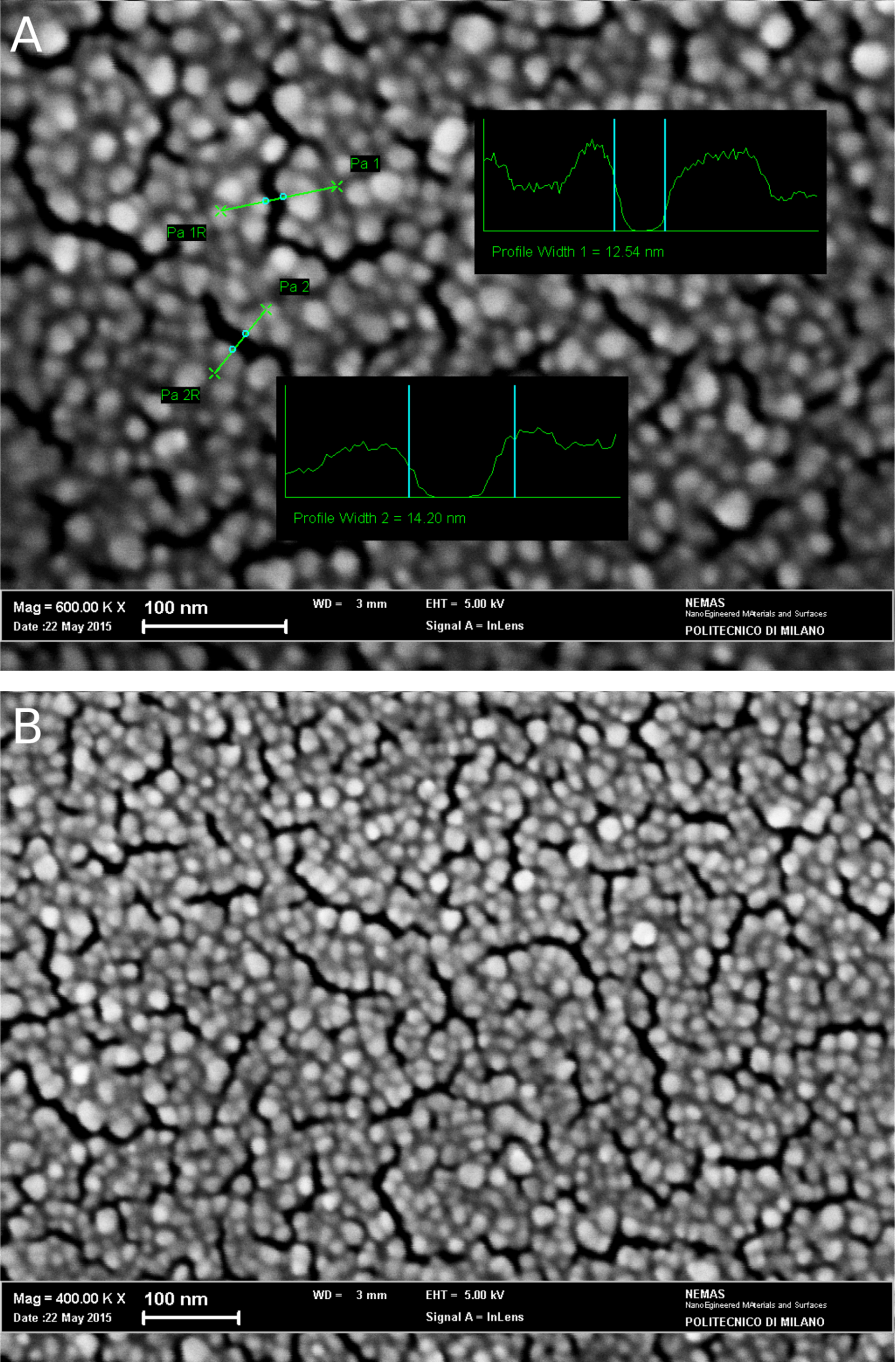
(A, B) SEM micrographs of the surface morphology of a gold substrate deposited at 100 Pa in Ar with 10^4^ laser pulses. Insets in (A) represent typical average sizes of the inter-island channels (*P*_a1_ = 12.54 nm and *P*_a2_ = 14.20 nm).

Figure 1 shows representative pictures of the surface morphology of the nanostructured gold substrates used as sensors for APO detection. The films consist of nearly spherical, mutually aggregated, Au NPs that give rise to islands with irregular shape, often interconnected with each other at specific sites. The channels with irregular shape and length separate adjacent islands. The insets of Figure 1A show typical average sizes of such inter-island channels. SERS hinges on the strong interaction between noble metal NPs and visible light through the resonant collective excitation of NP conduction electrons. The resulting surface plasmon resonance peak [28] displays a maximum at about 780 nm. This peak position is optimized by controlling the film nanostructure and allows the fluorescence to be kept as low as possible. No appreciable changes in the peak position and shape were observed after dipping the substrates in the solutions. The locally enhanced electric field decays abruptly within a tiny region of nanometer thickness around a given particle, with a marked dependence of the enhancement factor on the details of the local surface nano-roughness. The size of the inter-island channels makes them likely to have a twofold role. They are suitable to provide preferential, direct adsorption sites for the comparatively small molecular species to be studied. Here they can effectively interact with Au atoms and concurrently constitute hot spots where most of the relevant SERS enhancement occurs. This condition allows for the opportunity to optimize the nanostructure to be selective toward small species, such as drug molecules, with respect to a more bulky chemical species, for example, proteins in biological fluids. It is noteworthy that the above-discussed features of the artificially roughened surface in Figure 1A are homogeneous over areas of the order of 5 × 10^3^ nm^2^, as shown in Figure 1B. Such Au substrates are thus expected to display spatially uniform optical behavior. The typical enhancement factor determined for these substrates is 10^7^ [24].

### SERS spectra of APO aqueous solution

**Figure 2:**
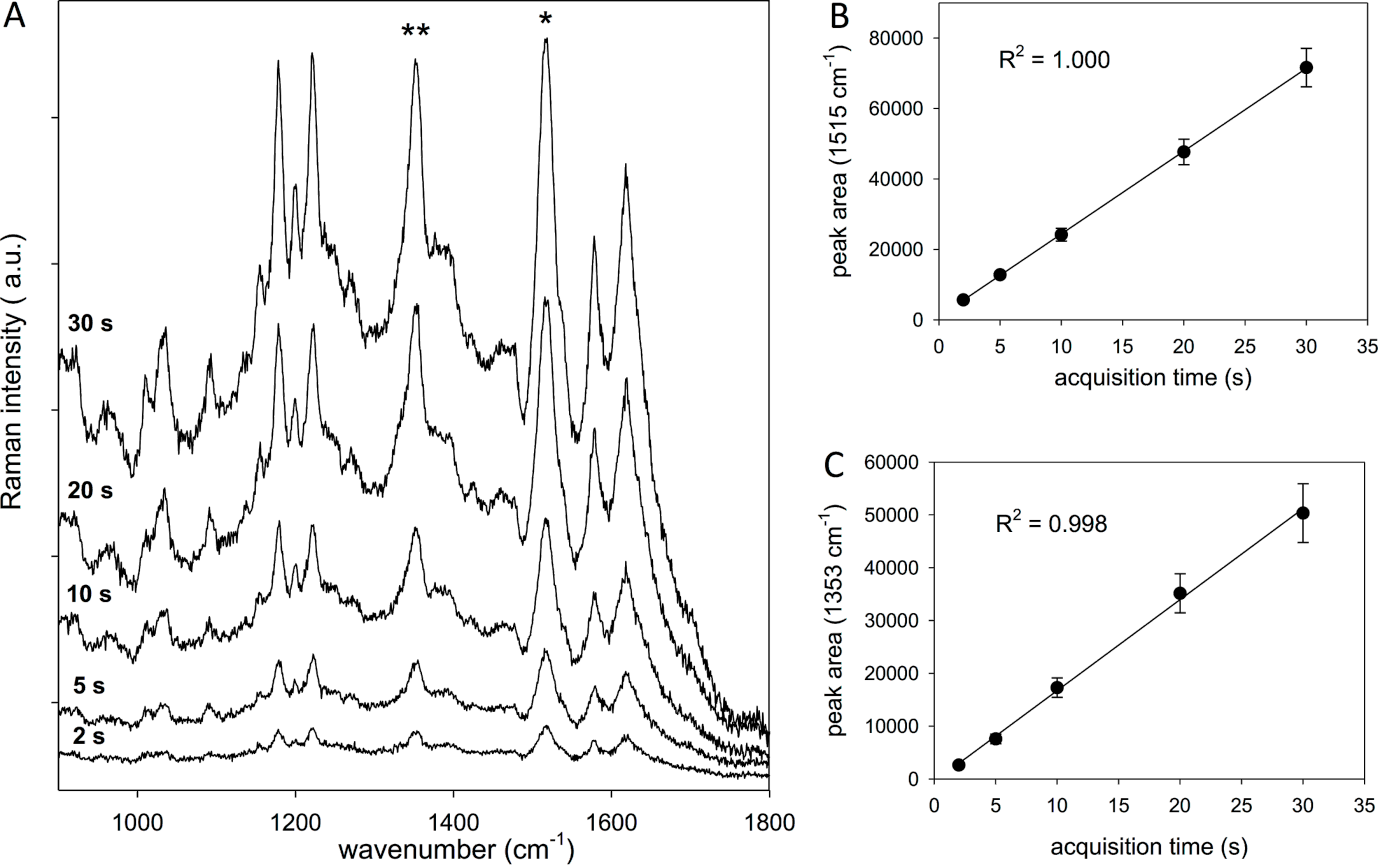
(A) SERS spectra of 100 µg/mL APO (dipping time: 5 min) collected with different exposure times on different areas of the gold substrate, and baseline-corrected peak areas versus acquisition time for two selected APO-intense Raman features at (B) 1515 cm^−1^ and (C) 1353 cm^−1^.

The SERS spectra of 100 µg/mL APO collected with different exposure times, ranging from 2 to 30 s, on different areas of the gold substrate are shown in Figure 2A. The substrate was dipped for 5 min in 1 mL of APO solution and air-dried before acquiring the SERS spectra.

By comparing the spectra, no meaningful changes in the spectral profile are observed during the measurements for the different exposure times. The selected experimental conditions (nominal laser power of 1 mW at the sample, spot size of about 1 µm, accumulation times of no longer than 30 s) are suitable to detect the analyte without producing any chemical degradation of the drug molecule during the measurements by interacting with the gold surface. The rapid acquisition time, together with a relatively low laser power, represent promising conditions for a rapid method for the measurement of the concentration of drugs in biological fluids. A spot size of about 1 µm ensures the collection of an average SERS signal from the selected area, given that inter-island channels of the nanostructured Au substrate are, on average, in the 10 nm range (see Figure 1).

The main SERS active modes of APO are found at 1009, 1035, 1091, 1179, 1199, 1223, 1353, 1392, 1515, 1579 and 1618 cm^−1^, in agreement with previous experimental and theoretical investigations [30].

The closeness of the molecule to the nanostructured metal surface is an inherent condition for SERS enhancement. Therefore, the assignment of experimental peaks to specific vibrational modes of the analyte is important to understand the kind of interaction between the molecule and the substrate. The assignment of the peaks also has a more practical meaning: it facilitates the selection and definition of appropriate spectral features that can be employed to recognize, and in some cases, to quantify, the analyte in progressively more complex matrices. The effort to resolve the analyte signals with respect to the overall spectrum, including contributions from interfering species, is a critical step once dealing with real samples.

Among the strongest peaks of the APO SERS spectrum, two signals were selected, specifically the peak centered at 1353 cm^−1^ (aromatic C–H in-plane bending, N–H bending, collective C–C stretching, CH_2_ wagging), and the one centered at 1515 cm^−1^ (C–C stretching of ring A, C–O stretching), in order to elucidate any dependence of the SERS signal on the experimental and instrumental parameters. Both wavenumbers fall in spectral regions where the SERS spectrum of blood plasma does not display any characteristic band. Using the near-infrared excitation at 785 nm, the spectra are dominated by protein bands with broad amide bands at 1650 cm^−1^ (amide I) and 1250 cm^−1^ (amide III), and sharp bands due to aromatic amino acids at 1004 cm^−1^ (Phe), 1127 cm^−1^ (Tyr) and CH_2_/CH_3_ deformations at 1450 cm^−1^ [31].

A manual baseline correction was applied to the raw data, and average values were plotted versus the acquisition time, as shown in Figure 2B. A linear regression was applied to the two data sets. The linearity of the SERS response confirms the observation that any of the selected acquisition times are suitable to detect intense APO signals, with no analyte degradation occurring during the measurement. This behavior also confirms the previously assessed [28–29] spatial reproducibility of the signal.

### Assessment of the concentration dynamic range of the sensor

To be considered a useful SERS-based method for drug detection, the procedure, based on the use of gold substrates pulsed laser deposition, should be rapid and quantitative. An acquisition time of 5 s, which corresponds to a fast measurement with an adequate signal-to-noise ratio (see Figure 2A), was chosen for the SERS measurements of APO aqueous solutions at different concentrations. To explore the dynamic range of the sensor, the Au SERS-active substrates were tested with respect to APO concentrations of 100, 10, 1 and 0.1 μg/mL.

**Figure 3:**
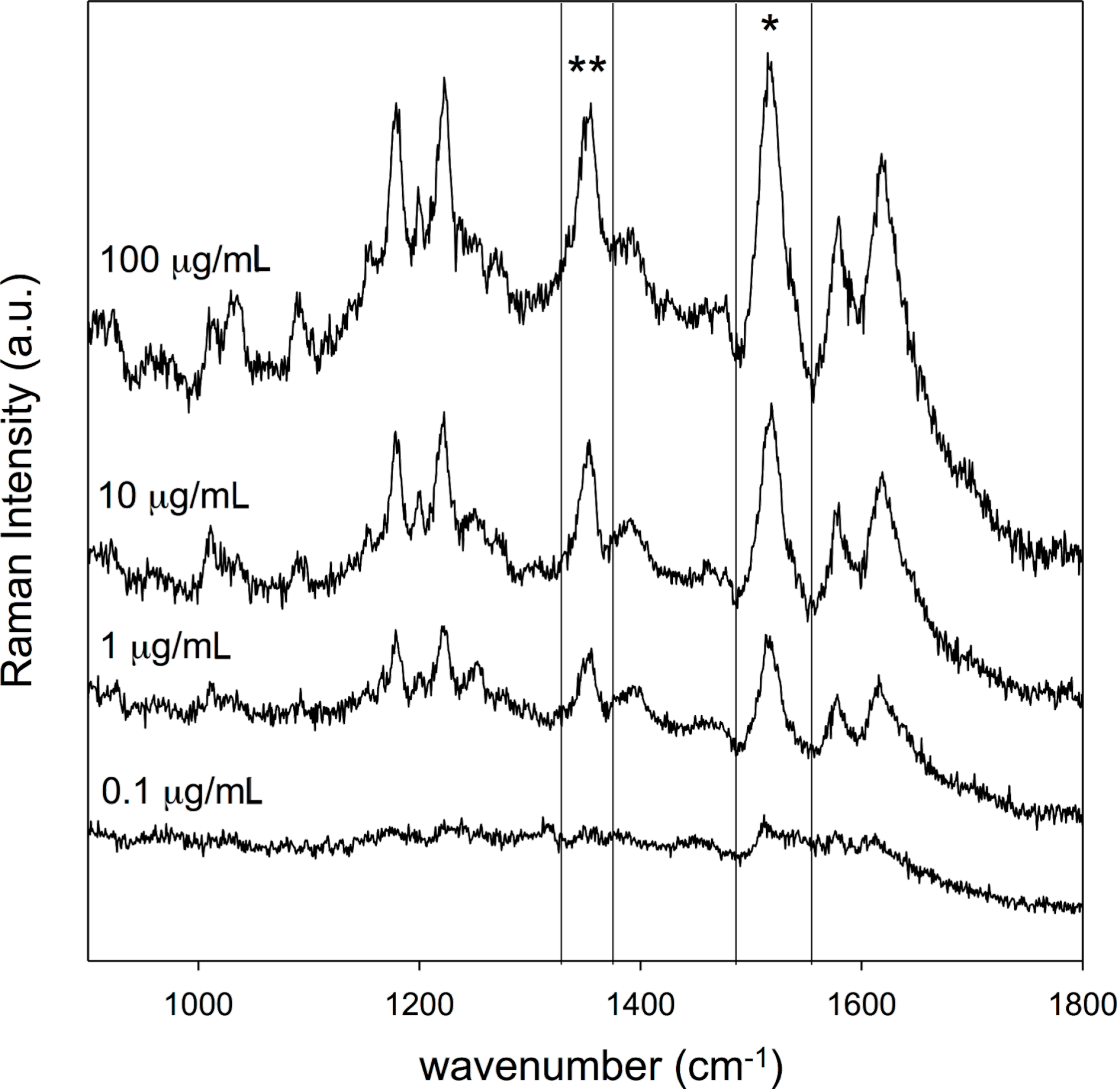
SERS of APO solutions at four different concentrations (100, 10, 1, 0.1 µg/mL; dipping time 5 min; 5 s acquisition time). The peaks at 1515 cm^−1^ and 1353 cm^−1^ were selected to evaluate the SERS signal dependence on the APO concentration.

Figure 3 shows a set of representative SERS spectra recorded with the above conditions. At the lowest concentration (0.1 µg/mL), only a weak signature at 1515 cm^−1^ is found. Different acquisition times, up to 30 s, did not result in an evident improvement in signal quality.

Starting from the concentration of 1 µg/mL, the intensity of APO peaks increases with APO concentration, while the position of all characteristic peaks remains unchanged. Dilution with water produces a change in the measured pH values of APO solutions from 5.5 for the most concentrated solution to 6.5 for the diluted solutions; such variations have no effect on the spectral features.

**Figure 4:**
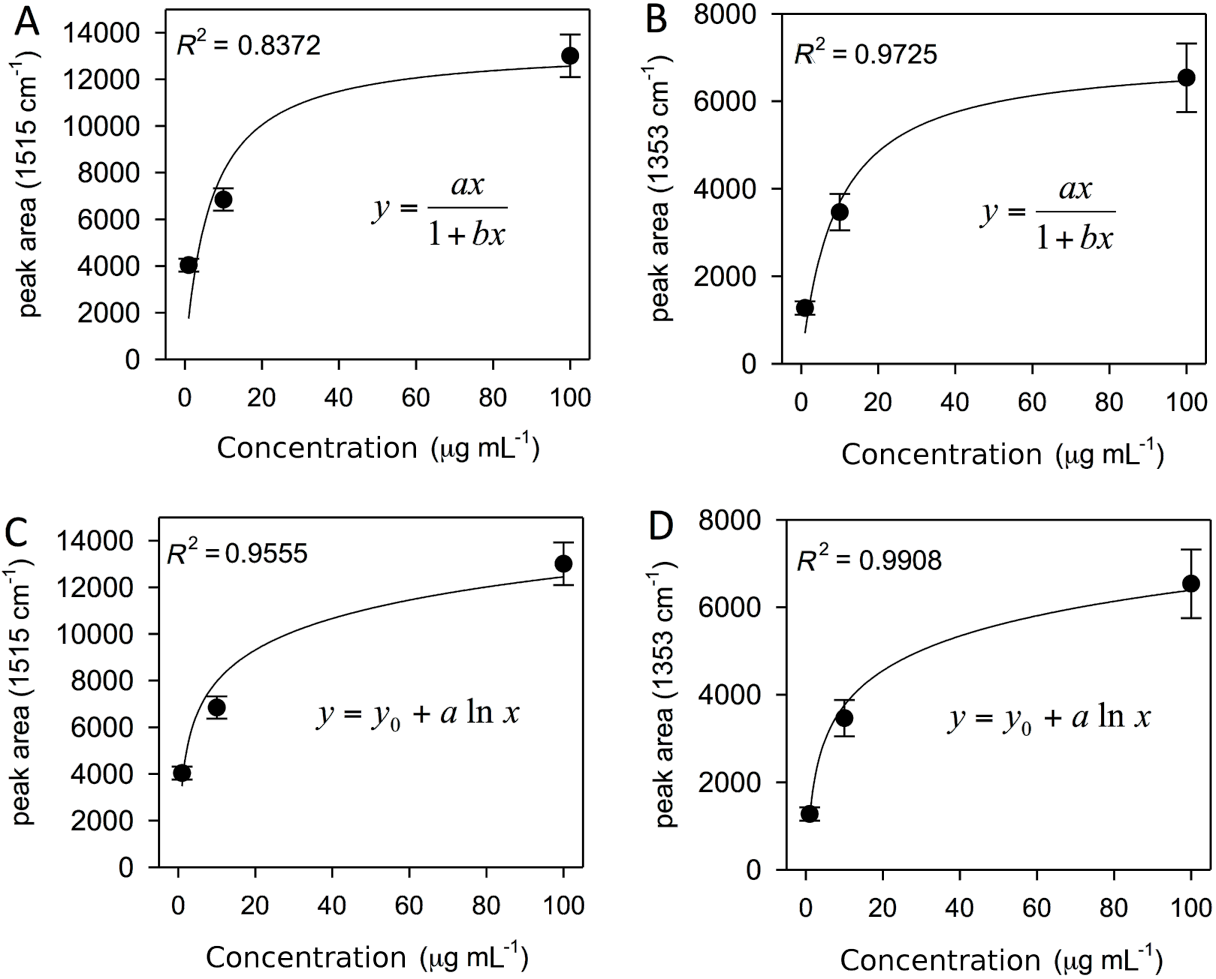
Peak areas (baseline-corrected) plotted against the concentration of APO solutions that summarize the dynamic concentration range of the substrate as a sensor for APO detection in aqueous solution. In particular (A) and (B) show a fit to a hyperbolic function of the data for 1515 cm^−1^ and 1353 cm^−1^ APO peaks; (C) and (D) show a fit to a logarithmic function of the data for the same peaks. The fitting parameters are (A) *a* = 2003, *b* = 0.15; (B) *a* = 773, *b* = 0.11; (C) *y*_0_ = 3476, *a* = 1948 and (D) *y*_0_ = 1125, *a* = 1143.

If the peak area is plotted against the concentration of the APO solutions, a characteristic trend is found, where a steep growth for low concentrations (between 1 and 10 µg/mL, notice that the lower limit falls within the range of clinical interest) and the onset of saturation near the highest tested concentration (100 µg/mL, see Figure 4) is observed.

These data points summarize quite well the whole dynamic range of concentrations of the sensor for APO detection in aqueous solution. The reduced number of selected concentrations allows for the exploration of the role of different experimental conditions on the sensor behavior in detail. For each point reported in Figure 4, a number of independent measurements (between 3 and 5) on a given sensor allowed the standard deviation to be estimated. This approach, together with the selected APO concentrations, is the best compromise to probe the dynamic range of the sensor and to test the repeatability of each measurement, while keeping a manageable number of sensors.

From the literature, different models were applied to fit the experimental data taking into account the nonlinearity of the curve trend when dealing with the quantitative application of SERS spectroscopy. The Langmuir isotherm was applied to qualitatively describe the concentration dependence and to find a nonlinear calibration curve for ten common narcotic drug analytes [32], while linear regression curves were generated from experimental data for glucose detection [33].

The coefficients of determination, *R*^2^, shown in Figure 4 indicate that a logarithmic model fits the experimental data slightly better than a hyperbolic model. The latter assumes an asymptotic limit (saturation), while the former allows the generation of linear regression curves when SERS peak areas for APO are plotted as a function of the logarithm of its concentration.

To evaluate the contribution of the dipping time to the definition of the dynamic range of the sensor, a shorter dipping time of 2 min (preferable for practical applications) was studied.

**Figure 5:**
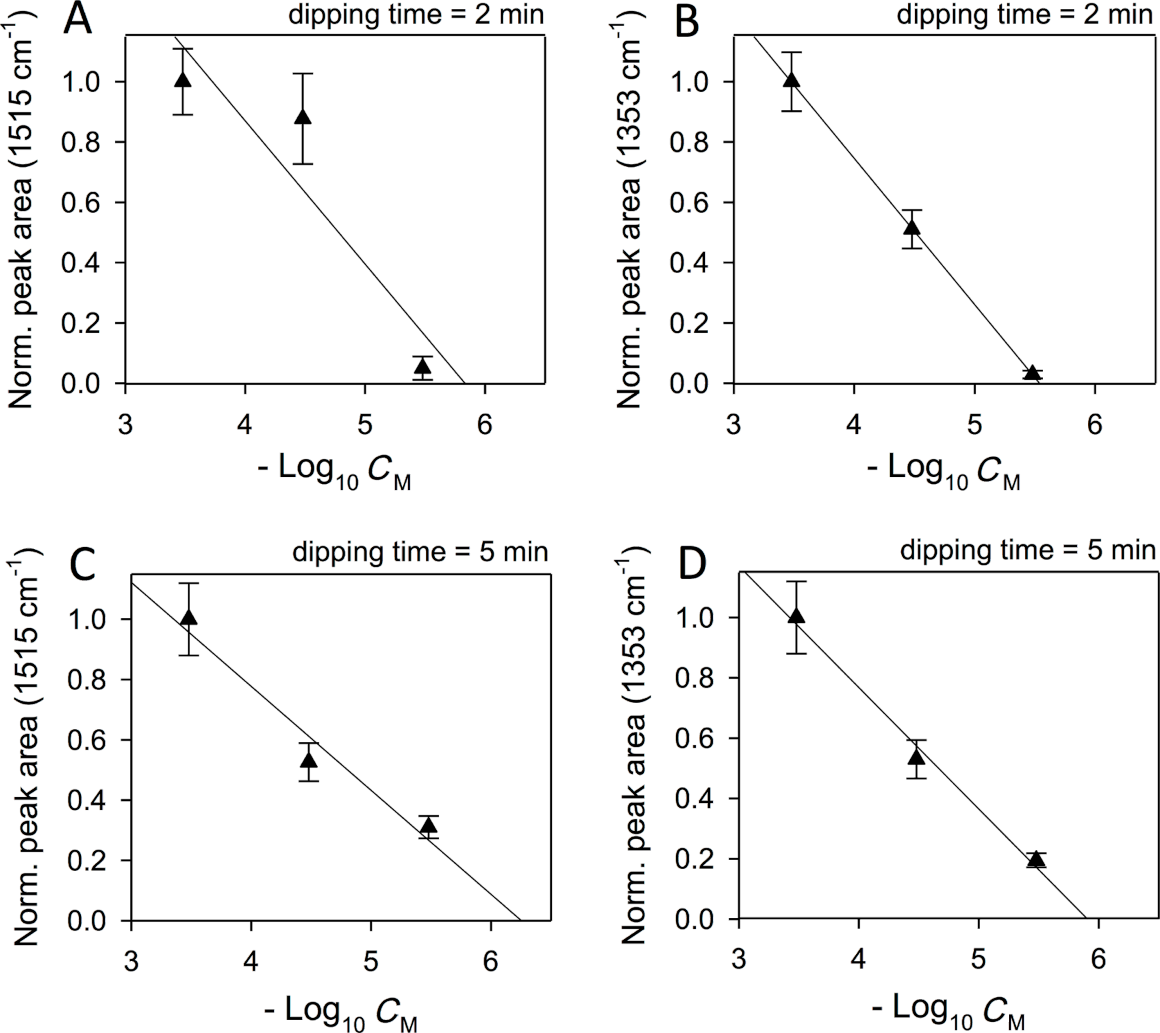
Effect of the dipping time on the dynamic range of the APO concentration of the gold sensor. Linearized data for different dipping times and APO peaks: (A) 2 min, 1515 cm^−1^; (B) 2 min, 1353 cm^−1^; (C) 5 min, 1515 cm^−1^; (D) 5 min, 1353 cm^−1^.

The SERS peak areas were normalized with respect to the peak area for the highest studied concentration (i.e., 100 µg/mL), and plotted on a semi-logarithmic scale (Figure 5). This way it is possible to contrast different curves obtained using different dipping times and to study the impact of this experimental parameter on the dynamic range.

A linear regression was applied to the data sets according to

[1]
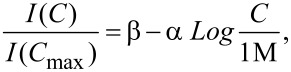


where *I*(*C*) is the Raman intensity of a selected APO peak, *C* is APO molar concentration and *C*_max_ = 3.3 × 10^−4^ M in our experiments; α and β are the fitting coefficients.

When the dipping time is 2 min, the slopes are α_1515_ = 0.5 ± 0.2 for the 1515 cm^−1^ peak, and α_1353_ = 0.486 ± 0.002 for the 1353 cm^−1^ peak; when the dipping time is 5 min, these become α_1515_ = 0.34 ± 0.07 and α_1353_ = 0.40 ± 0.04, respectively. Hence, the dipping time has a moderate role in the sensitivity of the sensor, and it could be conveniently optimized in order to reach the lowest detectable concentration for any chosen nanostructured, gold substrate. On the other hand, for a given dipping time, the α-parameter is constant with respect to the Raman mode. This is expected because the relative Raman intensity of two peaks reflects a molecular property that does not depend on concentration (i.e., it is a ratio of Raman cross-sections).

**Figure 6:**
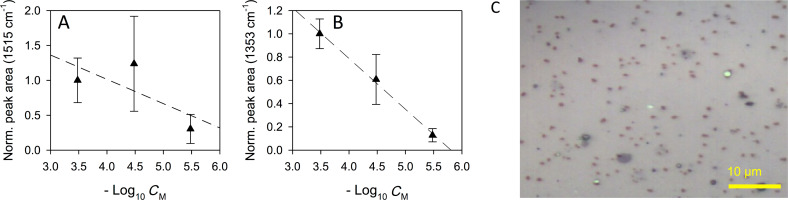
APO crystal growth on the gold sensor (dipping time 10 min). The scattered data result when plotting peak areas against APO concentration for (A) 1515 cm^−1^ peak and (B) 1353 cm^−1^ peak (see text). (C) Optical microscope image of a representative area of the sensor highlighting APO crystals (dark spots).

Based on these findings, an additional increase of the dipping time, while avoiding sensor saturation, could be expected to cause at least a slight sensitivity increase towards lower analyte concentrations. A third set of experiments at different APO concentrations was therefore carried out with a dipping time of 10 min. We observed the growth of APO crystals of a few micrometers on the surface of the sensor, irrespective of the concentration. More scattered data correspond to this observation (Figure 6). This limiting case should be avoided. On the contrary, optical microscope observation of the sensor surface did not reveal any distinct feature when the dipping time was 2 or 5 min. It is likely that the observed change in the sensor sensitivity on the dipping time is associated with changes in the degree of surface coverage.

### SERS measurements of APO in blood plasma

To prove the applicability of the proposed method to the detection and quantification of APO in blood samples, a few experiments were performed on unfiltered blood plasma with different APO concentrations. These challenging conditions were chosen to minimize sample preparation, while still demonstrating the feasibility of the proposed technique.

**Figure 7:**
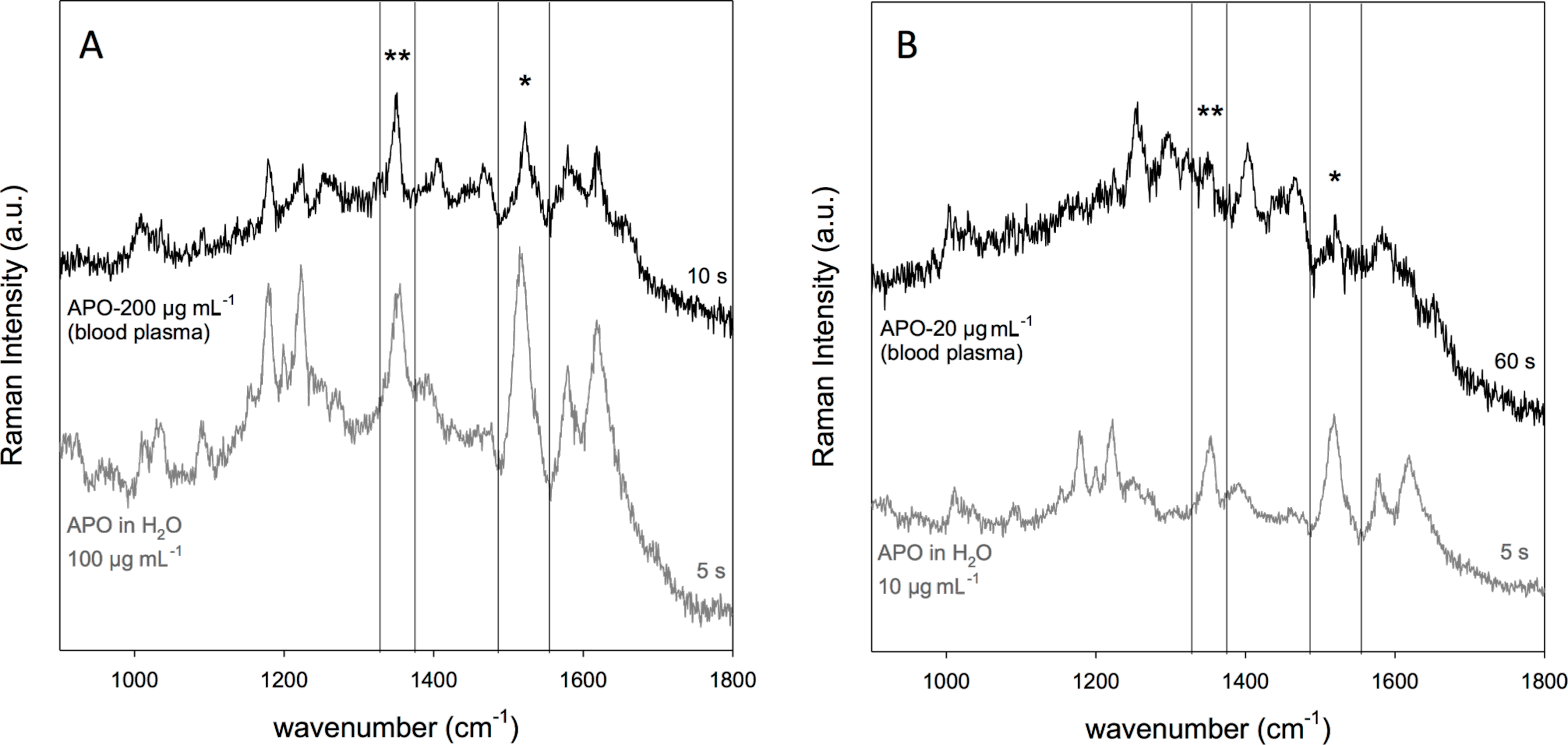
SERS spectra of unfiltered blood plasma with (A) APO 200 µg/mL, and (B) APO 20 µg/mL. SERS spectra of APO aqueous solutions (gray) are shown for comparison.

The SERS spectra are shown in Figure 7, where protein bands are noticeable at 1652 cm^−1^ (amide I), 1254 cm^−1^ (amide III), 1034 cm^−1^ and 1004 cm^−1^ (Phe).

In Figure 7A the SERS spectrum of 200 µg/mL APO in blood plasma shows intense peaks that can be ascribed to APO. Among these, the two peaks at 1515 and 1353 cm^−1^, selected for APO detection in aqueous solution, are clearly visible and found at 1520 and 1348 cm^−1^, respectively. Such differences in peak position can be possibly ascribed to the complexity of interactions that the drug molecule experiences in the biological matrix as compared to the aqueous solution. To reach a comparable SERS intensity with respect to 100 µg/mL APO in water, an acquisition time of 10 s was applied, while 60 s was the acquisition time required for a reasonable signal-to-noise ratio of the SERS spectrum of 20 µg/mL APO in blood plasma, as shown in Figure 7B.

The kinetics of APO adsorption onto the gold nanostructured surface are expected to be strongly affected by matrix effects. In particular, the presence of proteins, such as human serum albumin, an abundant plasma protein that binds a wide range of drugs including APO [28], has to be taken into account during the optimization of the experimental conditions. Hence, dipping times could be reconsidered to deal with the equilibrium between APO and proteins in solution.

Furthermore, once dealing with APO detection in clinical samples, it is possible to introduce minimized sample preparation (e.g., deproteinization) to enhance the APO signal intensity.

## Conclusion

Gold NP arrays pulsed laser deposited on glass proved to be suitable sensors for the quantitative detection of APO in water by means of SERS. The dynamic range of concentrations of the sensor was assessed by the selection of two SERS peaks. Minimal sample preparation and the capability to operate in an aqueous environment make the detection of APO by means of this technique rapid, with good signal-to-noise ratio, and characterized by adequate spatial reproducibility. The method is promising for transfer to APO detection in biological fluids. Experiments performed on unfiltered blood plasma with different APO concentrations proved the applicability of the proposed method to APO detection for samples of clinical origin.
